# Neoadjuvant strategies in resectable carcinoma esophagus: a meta-analysis of randomized trials

**DOI:** 10.1186/s12957-020-01830-x

**Published:** 2020-03-21

**Authors:** Tarun Kumar, Esha Pai, Rajesh Singh, Neville J. Francis, Manoj Pandey

**Affiliations:** 1 Department of Surgical Oncology, Banaras Hindu University, Varanasi, 221005 India; 2 Department of Surgical Oncology, Tata Memorial Centre, Mumbai, 400012 India; 3 Department of Surgical Oncology, Asian Institute of Oncology, Mumbai, 400022 India

**Keywords:** Neoadjuvant chemotherapy, Neoadjuvant chemoradiation, Neoadjuvant radiation, Sequential chemoradiation, Carcinoma esophagus, Preoperative chemotherapy, Preoperative chemoradiation

## Abstract

**Background:**

The survival benefit of neoadjuvant therapy in resectable carcinoma esophagus has been elucidated. We performed a meta-analysis in light of new studies and long-term results of past trials. The search strategy was refined to include only “neoadjuvant” so that any bias by adjuvant treatment is eliminated.

**Methods:**

A detailed search of MEDLINE, Embase, and Cochrane Library was done. Only published randomized English language trials were included. Data were categorized as neoadjuvant concurrent chemoradiation (NACRT), neoadjuvant chemotherapy (NACT), neoadjuvant radiotherapy (NART), and neoadjuvant sequential chemoradiotherapy (SCRT). Meta-analysis was done using odds ratio (OR) and 95% CI using fixed/random effects model. Heterogeneity was tested by chi-square and *I*^2^ test. *Z* probability calculated significant difference across subgroups. Outcomes assessed were overall survival (OS) and disease-free survival (DFS) at 3 and 5 years, respectively, mortality (30/90 day) and failures (local/systemic).

**Results:**

Twenty-five randomized trials involving 5272 patients were included for quantitative analysis. NACRT was evaluated in 12 studies (2676 patients). Superior 3-year OS (OR = 0.68 CI 0.52–0.90, *p* = 0.007), 3-year DFS (OR = 0.55 CI 0.45–0.68, *p* = 0.00001), and 5-year DFS (OR = 0.59 CI 0.47–0.74, *p* = 0.00001), with lower failures (OR = 0.52 CI 0.37–0.73, *p* = 0.0001), were seen in favor of NACRT at the cost of increased perioperative mortality (OR = 1.79 CI 1.15–2.80, *p* = .01). However, 5-year OS (OR = 0.78 CI 0.60–0.1.01, *p* = 0.06) was not found to be significantly superior. NACT, NART, and SCRT were not found to have any benefit over surgery alone.

**Conclusion:**

This meta-analysis presents strong evidence favoring NACRT over upfront surgery. It also shows no survival advantage of neoadjuvant chemotherapy.

## Introduction

The outcomes of resectable esophageal cancer have remained dismal with an estimated 5-year survival of 50–55% [1]. To overcome this, a number of studies were carried out to evaluate the impact of the addition of chemotherapy and radiotherapy to surgery [2–27]. These trials evaluated the effect of adjuvant and neoadjuvant treatment but with equivocal results. Adjuvant therapy merits an accurate pathological staging of disease but also risks non-completion of systemic therapy in the face of surgical morbidity. Meanwhile, neoadjuvant treatment has its advantages in the form of undisturbed tumor bed with better oxygenation and optimal patient performance in a treatment-naive setting but at the cost of increased perioperative morbidity and mortality.

Neoadjuvant treatment of esophageal cancer, in the recent past, has drawn attention with many newer trials having been published [11, 16]. With inconclusive evidence [28–30] comparing neoadjuvant chemoradiotherapy (NACRT) to neoadjuvant chemotherapy (NACT) and meta-analyses [31] not showing convincing results favoring either modality of treatment, NACRT and NACT, continue to be practiced in different parts of the world as per institutional protocol and individual preferences.

We, in this meta-analysis, have compared NACRT and NACT separately to upfront surgery with a more refined and stringent search strategy to elucidate any possible effect that either may have. We also included the newest randomized trial [11] and long-term results of previously published trial [16]. Prior meta-analyses [31–33] on this subject have considered studies where adjuvant treatment was given (chemotherapy or radiotherapy) after surgery following neoadjuvant treatment, making it difficult to interpret the impact of purely neoadjuvant treatment. Hence, we included randomized control trials where only neoadjuvant treatment strategies were used without any peri- or post-operative treatment, so that any bias introduced by adjuvant treatment may be eliminated. This meta-analysis uses only published randomized controlled trials. Other unpublished abstracts presented in meetings and prospective cohort studies have not been included.

## Methods

We included all the published randomized controlled trials of neoadjuvant treatment for esophagus or esophago-gastric cancer (EGC) (Siewert 1 and 2) followed by surgery versus upfront surgery for resectable disease. A detailed literature search was carried out in MEDLINE (PubMed), Embase, and Cochrane databases. We used the following search strings:

(("esophagus"[All Fields] OR "esophagus"[MeSH Terms] OR "esophagus"[All Fields]) OR ("esophagus"[All Fields] OR "esophagus"[MeSH Terms] OR "esophagus"[All Fields])) AND (("neoadjuvant therapy"[MeSH Terms] OR ("neoadjuvant"[All Fields] AND "therapy"[All Fields]) OR "neoadjuvant therapy"[All Fields] OR "neoadjuvant"[All Fields]) OR preoperative[All Fields]) AND (("drug therapy"[Subheading] OR ("drug"[All Fields] AND "therapy"[All Fields]) OR "drug therapy"[All Fields] OR "chemotherapy"[All Fields] OR "drug therapy"[MeSH Terms] OR ("drug"[All Fields] AND "therapy"[All Fields]) OR "chemotherapy"[All Fields]) OR ("radiotherapy"[Subheading] OR "radiotherapy"[All Fields] OR "radiotherapy"[MeSH Terms]) OR ("chemoradiotherapy"[MeSH Terms] OR "chemoradiotherapy"[All Fields])) AND Clinical Trial[ptyp] AND English[lang]. The last search was done on August 28, 2019, on PubMed.

**Fig. 1:**
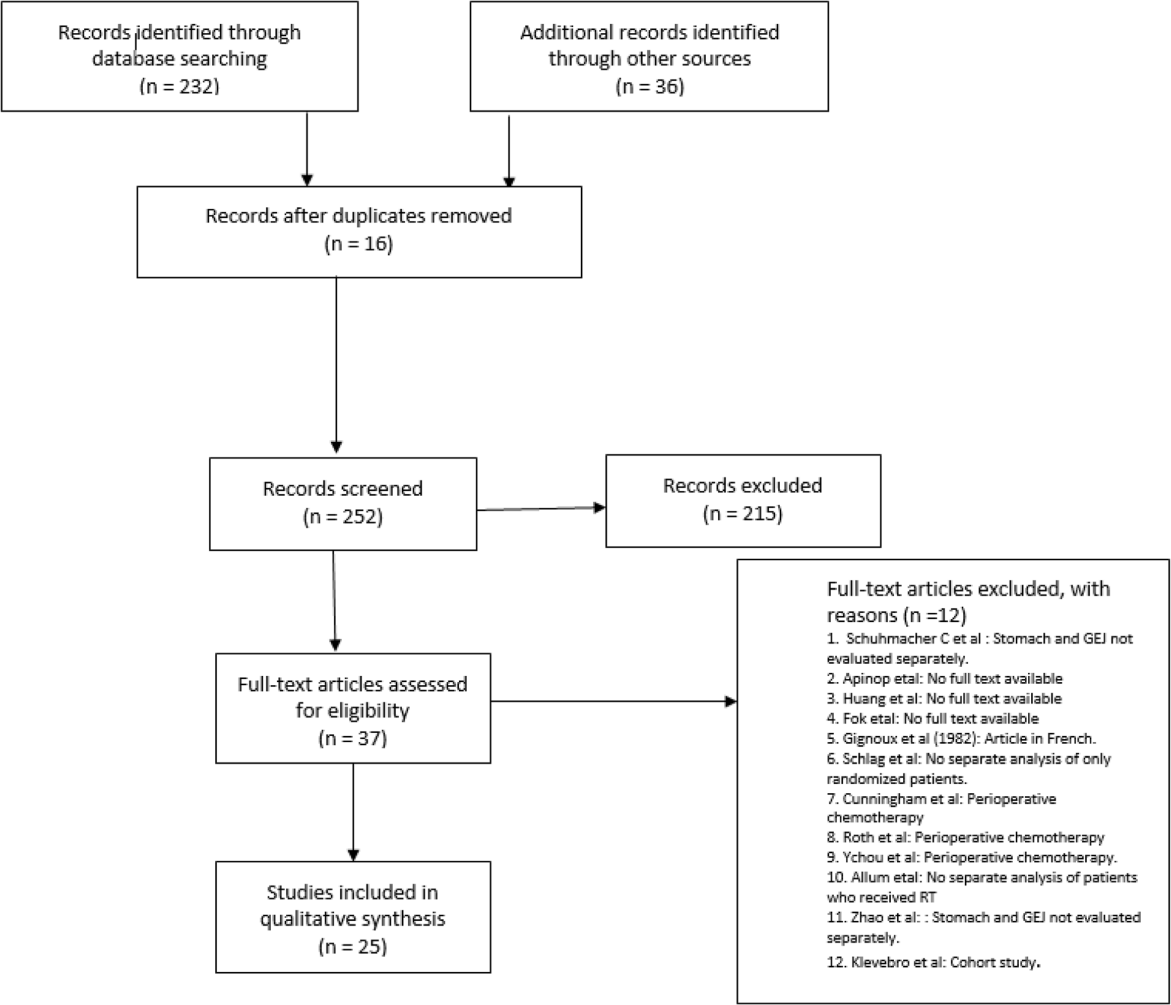
PRISMA flowchart

Out of 232 studies found from the above search, 16 were shortlisted after abstract reviews. An additional 33 studies were identified from previous meta-analyses and 3 were found during a manual search of back-references. In all, after the elimination of duplicates and exclusions, 25 studies were considered for the final analysis (Fig. 1, Table 1).

**Table 1:** The quality of each study evaluated using Jadad’s score

	Study and reference no.	Arms	Sample size	Treatment schedule	No. of cycles	Jadad’s score			
						*R*	*B*	*D*	Total
1.	Ancona et al. (2001) [3]	NACT vs Sx	96	Cisplatin 100 mg/m^2^on day 1 5-Fluorouracil 1000 mg/m^2^ days 1 to 5	02 (21-day cycle)	1 + 1	0	1	3
2.	Kelson et al. (1998) [4]	NACT vs Sx	440	Cisplatin 100 mg/m^2^ on day 1 5-Fluorouracil 1000 mg/m^2^ days 1 to 5	03 (29-day cycle)	1 + 1	0	1	3
3.	Law et al. (1997) [5]	NACT vs Sx	147	Cisplatin 100 mg/m^2^ on day 1 5-Fluorouracil 1000 mg/m^2^ days 1 to 5	02 (22–26-day cycle)	1	0	1	2
4.	MRC (2002) [9]	NACT vs Sx	802	Cisplatin 100 mg/m^2^ on day 1 5-Fluorouracil 1000 mg/m^2^ days 1 to 4	02 (21-day cycle)	1 + 1	0	1	3
5.	Maipang et al. (1994) [7]	NACT vs Sx	46	Cisplatin 100 mg/m^2^ on day 1 Vinblastine, 3 mg/m^2^ on days 1, 8, 15, and 22 Bleomycin 10 mg/m^2^, on day 3 followed by a 4-day infusion of 10 mg/m^2^/day	02 (29-day cycle)	1	0	1	2
6.	Boonstra et al. (2011) [23]	NACT vs Sx	169	Cisplatin 100 mg/m^2^ on day 1, Etoposide 100 mg/m^2^ intravenously on days 1 and 2, followed by Etoposide 200 mg/m^2^ orally on days 3–5.	03 (28-day cycle)	1	0	1	2
7.	Baba et al. (2000) [22]	NACT vs Sx	21	Cisplatin 70 mg/m^2^on day 1 5-Fluorouracil 700 mg/m^2^ days 1 to 5 Leucovorin 20 mg/m^2^days 1 to 5	02 (28-32 day cycle)	1 + 1	0	1	3
8.	Bosset et al. (1997) [24]	NACRT vs Sx	282	Cisplatin 80 mg/m^2^on days 0–2 Total radiation dose: 37.5 Gy	02	1	0	1	2
9.	Urba et al. (2001) [18]	NACRT vs Sx	100	Cisplatin 20 mg/m^2^ days 1–5 5-Fluorouracil 300 mg/m^2^ days 1–21 Vinblastine 1 mg/m^2^ days 1–4 Total radiation dose: 45 Gy	02	1	0	1	2
10.	NEOCTREC (2018) [11]	NACRT vs Sx	451	Cisplatin 75 mg/m^2^ day 1 or Cisplatin 25 mg/m^2^ days 1 to 4 Vinorelbine 25 mg/m^2^days 1 and 8 Total radiation dose: 40 Gy	02	1 + 1	0	1	
11.	Lee et al. (2004) [6]	NACRT vs Sx	101	Cisplatin 60 mg/m^2^day 1 5-Fluorouracil 1000 mg/m^2^ days 2 to 5 Total radiation dose: 45.6 Gy	02	1 + 1	0	1	3
12.	Shapiro et al. (2015) [16]	NACRT vs Sx	366	Carboplatin AUC 2 mg/ml/min day 1 Paclitaxel 50 mg/m^2^ day 1 Total radiation dose: 41.4 Gy	05	1 + 1	0	1	3
13.	Natsugoe et al. (2006) [10]	NACRT vs Sx	45	Cisplatin 7 mg 5-Fluorouracil 350 mg Total radiation dose: 40 Gy	20	1 + 1	0	1	3
14.	Tepper et al. (2008) [17]	NACRT vs Sx	56	Cisplatin 100 mg/m^2^ day 1 5-Fluorouracil 1000 mg/m^2^ days 1–4 Total radiation dose: 50.4 Gy	02	1	0	1	2
15.	Mariette et al. (2014) [8]	NACRT vs Sx	195	Cisplatin 75 mg/m^2^ day 1 5-Fluorouracil 800 mg/m^2^ days 1–4 Total radiation dose: 45 Gy	02	1 + 1	0	1	3
16.	Lv et al. (2010) [34]	NACRT vs Sx	238	Cisplatin20 mg/m^2^days 1–3 Paclitaxel 135 mg/m^2^ day 1 Total radiation dose: 40 Gy	02	1 + 1	0	0	2
17.	Walsh et al. (1996) [19]	NACRT vs Sx	113	Cisplatin75 mg/m^2^ day 7 5-Fluorouracil 15 mg/kg/day days 1–5 Total radiation dose: 40 Gy	02	1	0	1	2
18.	Burmeister et al. (2005) [25]	NACRT vs Sx	256	Cisplatin 80 mg/m^2^ 5-Fluorouracil 800 mg/m^2^ days 1–4 Total radiation dose: 35 Gy	01	1 + 1	0	1	3
19.	Le Prise et al. (1994) [13]	SCRT vs Sx	104	Cisplatin 100 mg/m^2^ day 1 5-Fluorouracil 600 mg/m^2^ days 2–5 Total radiation dose: 20 Gy	02	0	0	1	1
20.	Wang et al. (1998) [20]	NART vs Sx	206	Total radiation dose: 40 Gy		1	0	0	1
21.	Lanouis et al. (1981) [35]	NART vs Sx	124	Total radiation dose: 40 Gy		1	0	1	2
22.	Gignoux et al. (1987) [27]	NART vs Sx	126	Total radiation dose: 33 Gy		1	0	0	1
23.	Arnott et al. (1992) [21]	NART vs Sx	129	Total radiation dose: 20 Gy		1 + 1	0	1	3
24.	Nygaard et al. (1992) [12]	NACT vs SCRT vs NART vs Sx	186	Cisplatin 20 mg/m^2^days 1 to 5 Bleomycin 50 mg/m^2^ days 1–5 Total radiation dose: 35	02 (15–23-day cycle)	1	0	1	2
25.	Cao et al. (2009) [26]	NACT vs NACRT vs NART vs Sx	473	Cisplatin 20 mg/m^2^ days 1–5 5-Fluorouracil 500 mg/m^2^ days 1–5 Mitomycin 10 mg/m [2]/day, day 1 Total radiation dose: 40 Gy	NA	1	0	0	1

*NACT* neoadjuvant chemotherapy, *NACRT* neoadjuvant concurrent chemoradiation, *NART* neoadjuvant chemoradiation, *SCRT* neoadjuvant sequential chemoradiation, *Sx* surgery, *R* randomization, *B* blinding, *D* description

## Inclusion and exclusion criteria

All randomized trials that compared upfront surgery with neoadjuvant chemotherapy (NACT), radiotherapy (NART), concurrent chemoradiation (NACRT), or sequential chemoradiation (SCRT) followed by surgery were included. Operability was considered based on preoperative evaluation techniques of respective studies based on tumor size, extent, infiltration into surrounding tissues, lymph node, or distant metastases as evaluated by the authors of individual trials, respectively.

Exclusion criteria entailed perioperative chemotherapy, where adjuvant treatment (chemotherapy or radiation) was given, mixed tumor subsites (stomach and esophagus) where subset analyses of esophageal and gastric cancers were not done separately, and unpublished abstracts presented in meetings and in non-English language manuscripts.

### Data extraction

Two authors individually scanned all abstracts and shortlisted studies meeting the above inclusion criteria. Data from each of these shortlisted studies was collected on a pre-set proforma. Any discrepancies in the inclusion were settled after discussion with a third member. The quality of each study was evaluated using Jadad’s score [36] (Table 1). Data were categorized into four groups: neoadjuvant concurrent chemoradiation, neoadjuvant chemotherapy, neoadjuvant radiotherapy, and neoadjuvant sequential chemoradiation.

For final analyses, 6 endpoint variables were compared. The primary endpoints were overall survival (OS) and disease-free survival (DFS) at 3 and 5 years. Secondary endpoints were 30- or 90-day mortality (as given in the studies) and failures (local and systemic). The OS is defined as the time from date of recruitment to death, and DFS was described as time from recruitment to recurrence or metastasis, as described in studies. Publication bias was measured using a funnel plot [37].

### Statistical analysis

Treatment failure rates, mortality rates, 3- and 5-year overall and disease-free survival rates were extracted and entered in the REVMAN 5.2 software. For dichotomous variables, odds ratios with their 95% confidence intervals were calculated with Mantel-Haenszel methods using fixed (if no heterogeneity) or random-effects model (*I*^2^ > 40%) [38]. Heterogeneity was tested using chi-square and *I*^2^ tests, and significance was tested using the *Z* test.

The manuscript has been presented in accordance with the Preferred Reporting Items for Systematic Reviews and Meta-Analyses (PRISMA) guidelines [39], and the checklist of the same has been provided in the supplementary material (Supplementary file 15).

## Results

Twenty-five randomized trials involving 5272 patients were included for quantitative analysis. All variables were not available in all studies, hence only those studies with particular variables were used for individual meta-analysis.

### Neoadjuvant chemoradiotherapy (NACRT)

**Fig. 2:**
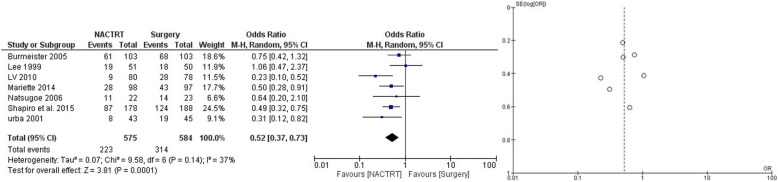
Forest and funnel plot comparing failures in NACRT and upfront surgery arms

**Fig. 3:**
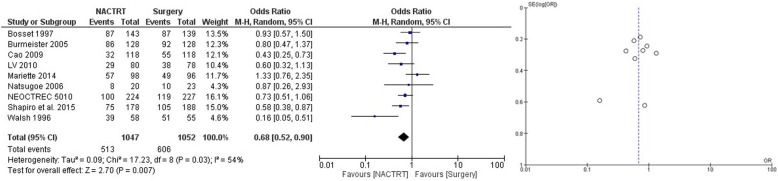
Forest and funnel plot comparing OS at 3 years in NACRT and upfront surgery arms

**Fig. 4:**
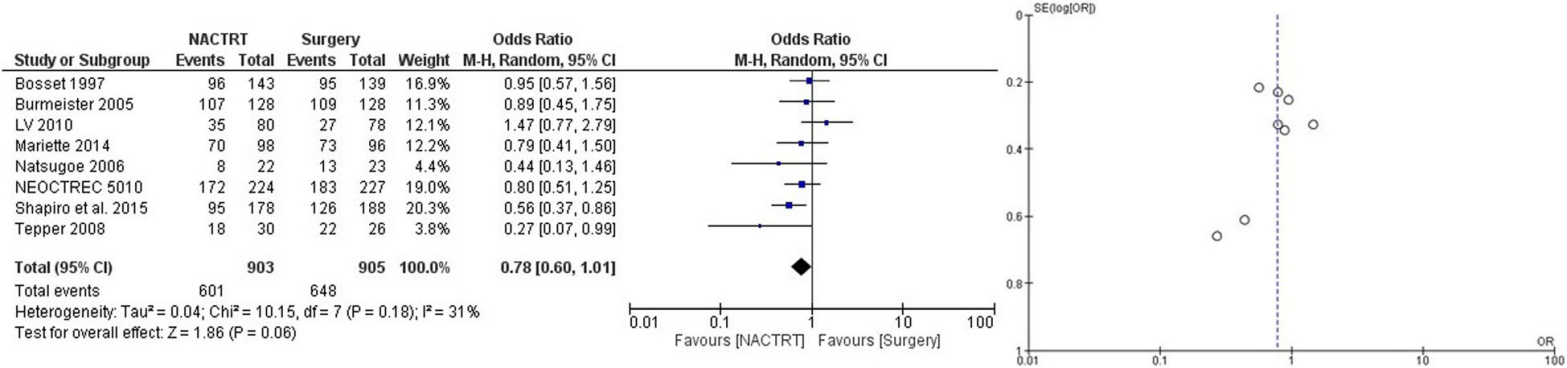
Forest and funnel plot comparing OS at 5 years in NACRT and upfront surgery arms

**Fig. 5:**
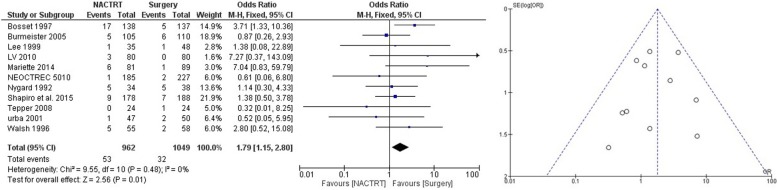
Forest and funnel plot comparing perioperative mortality in NACRT and upfront surgery arms

NACRT was the most extensively studied neoadjuvant strategy with a total of 12 trials and 2676 patients. The results showed distinct advantage of NACRT over upfront surgery with lower failures (local and systemic) (OR = 0.52 CI 0.37–0.73, *p* = 0.0001) (Fig. 2), superior DFS at 3 years (OR = 0.55 CI 0.45–0.68, *p* = 0.00001) (Supplementary file 1), superior DFS at 5 years (OR = 0.59 CI 0.47–0.74, *p* = 0.00001) (Supplementary file 2), and superior OS at 3 years (OR = 0.68 CI 0.52–0.90, *p* = 0.007) (Fig. 3). There was a trend towards survival benefit seen in OS at 5 years (OR = 0.78 CI 0.60–0.1.01, *p* = 0.06) (Fig. 4); however, it did not reach significance. Significantly more perioperative mortality was seen (30 or 90 days) in the NACRT group (OR = 1.79 CI 1.15–2.80, *p* = .01) (Fig. 5) compared to the surgery-alone arm.

### Neoadjuvant chemotherapy (NACT)

NACT was evaluated in nine studies with a total of 2380 patients. NACT followed by surgery failed to show any significant benefit over upfront surgery alone for any of the variables studied. There was a trend towards superior 3-year OS (OR = 0.85, 95% CI 0.68–1.06, *p* = 0.14) (Supplementary file 6) and 5-year OS (OR = 0.72 95% CI 0.49–1.07, *p* = 0.11) (Supplementary file 8) in favor of NACT, but it failed to reach statistical significance.

### Neoadjuvant radiotherapy (NART)

NART was investigated in 6 trials with a total of 1244 patients. Treatment-related mortality for neoadjuvant radiotherapy was studied in 3 trials with 439 participants and showed no significant difference (Supplementary file 9). The 3-year OS was reported in 3 trials, with a total of 865 patients whereas 5-year OS was reported in 4 trials with 582 patients. There was a distinct 42% benefit in the 3-year OS (OR = 0.58 95% CI 0.39–0.86, *p* = 0.007) (Supplementary file 11) in favor of NART followed by surgery which was inapparent in the 5-year OS (OR = 1.01 95% CI 0.65–1.59, *p* = 0.96) (Supplementary file 12).

### Sequential chemoradiation (SCRT)

There were 2 studies with 290 patients which investigated SCRT. Only two variables, i.e., perioperative mortality and 3-year OS were recorded in these studies (supplementary material 13 & 14). The results of neither of the two variables reached clinical significance.

## Discussion

This meta-analysis demonstrates the benefit of NACRT across all variables evaluated, except 30/90-day mortality. NACRT showed a significant overall survival benefit of 32% at 3 years. This benefit diminished at 5 years (22%) as it only showed a trend towards statistical significance. However, these figures need to be interpreted with caution as the studies had heterogeneity amongst them. Further, these promising results were at the cost of a 79% increase in perioperative mortality in the NACRT group compared to surgery alone. The meta-analysis also showed 48% lower recurrences (systemic and locoregional) in the NACRT arm. In comparison, Chan et al. [33] showed an absolute survival benefit of 25% with a 46% increase in perioperative mortality with NACRT whereas Sjoquist and colleagues [31] showed an absolute benefit of 8.7% in all-cause mortality at 2 years.

The 5-year OS did show a strong trend towards survival benefit but did not reach significance and can be interpreted in three disparate ways. First, we hypothesize lack of sufficient data, i.e., if there was probably one more trial or more patients in any of the trials wherein survival in the NACRT group was higher, and this figure would also be significant like all the other variables. Second, NACRT did not fare better than upfront surgery as some of the long-term failures of surgery alone may have been salvaged with radiation or chemotherapy so that the 5-year OS was similar to that of NACRT; however, this information on the use of salvage therapy is missing in most studies. Third, there is also a possibility of increased non-cancer-related deaths in the long term in the NACRT group. The latter was seen in the NeoRes trial by Klevebro et al. [30] which demonstrated 46% of NACRT patients died of non-cancer-related deaths at 1 year. With the data available to us, it is not possible to comment on this phenomenon, and it should be seen as an absence of evidence and not as evidence of absence.

Another point of contention is whether the perioperative mortality in these trials has been included as events in the calculation of OS for the NACRT arm. None of the trials have explicitly described this pertinent aspect in their articles, and it is not clear if they have used an intention-to-treat analysis, hence the exact statistical effect of perioperative mortality on overall survival cannot be ascertained.

All these results are not surprising as the comparison is being made between two unbalanced arms, i.e., multimodality treatment (radiotherapy, chemotherapy, surgery) versus single modality (surgery alone). It is now established that surgery alone is insufficient for the treatment of resectable esophageal cancer and the addition of chemoradiotherapy is essential to achieve better results. However, the timing of the chemoradiotherapy (preoperative or postoperative) needs to be investigated as there may be a reduction in peri-operative mortality if chemoradiotherapy is given in the adjuvant setting, instead of as neoadjuvant. The QUINTETT study by Malthaner et al. [40] presented as an abstract compared health-related quality of life (HRQL) of neoadjuvant and adjuvant chemoradiation for resectable esophageal cancer. They showed no significant difference in HRQL scores but reported significantly more chemotherapy-related adverse events in the neoadjuvant arm. Surgery-related adverse events were also significantly more in the neoadjuvant arm. Miccio et al. [41] analyzed the surveillance, epidemiology, and end result (SEER) registry database from 2001–2011 of Siewert’s type II GEJ cancers. Of 1497 patients they analyzed, 746 received adjuvant RT and 751 received neoadjuvant RT. They concluded that adjuvant RT is associated with a significantly lower death risk hazard ratio (0.84 95%CI 0.73–97, *p* = 0.016) and disease-specific death risk hazard ratio (0.84 95%CI 0.72–0.97, *p* = .02). On multivariate analysis in an epidemiological study by Nassri et al. [42], it was shown that the only significant factor which benefits survival is esophagectomy, i.e., the surgery itself. They reported that 16% of patients on neoadjuvant treatment had disease progression, and only 41% underwent definitive surgery. However, the resectability rates in other studies have been quoted to be higher. On the contrary, there is retrospective data [43] suggesting that NACRT does not increase any postoperative complications with a trend towards survival benefit. A phase II randomized trial by Mao et al. (NCT01463501) which is prospectively studying the difference between neoadjuvant and adjuvant paclitaxel and carboplatin-based chemoradiation in terms of quality of life and adverse events as a measure of safety and tolerability is underway and its results are long-awaited. However, the CROSS trial by Van Hagen et al. [44] showed 4% mortality in each of the NACRT and upfront surgery arms.

NACT followed by surgery has been a popular treatment strategy practiced widely. However, this meta-analysis did not find a significant advantage of NACT in terms of OS, DFS, locoregional/systemic failures, or perioperative mortality. This is in contrast to the previous two meta-analyses by Gebski et al. [32] and Sjoquist et al. [31] which demonstrated an absolute survival benefit of 7% and 5.1%, respectively, at 2 years, with NACT. Chan et al. [33] also showed a trend towards survival benefit with NACT, but this did not reach statistical significance. The superior results attributed to “NACT” in these studies were probably due to the benefit conferred by perioperative chemotherapy. Former meta-analyses included trials [45–47], which administered perioperative chemotherapy (preoperative and postoperative chemotherapy), thus explaining the survival benefit seen. These “peri-operative” chemotherapy studies were excluded from our analysis, and hence, our study reports the role of chemotherapy in a purely neoadjuvant sense, finding no benefit whatsoever.

NART followed by surgery made a significant impact on a 3-year OS, reducing the risk by 42% (OR = 0.58 CI 0.39–0.86, *p* = .007) which continued to show a trend towards significance at 5 years. Perioperative mortality and failures were not significantly different in the two groups. This strategy has not been extensively evaluated and no trials have compared chemoradiotherapy (CTRT) with radiotherapy (RT) alone.

The two trials that studied SCRT did not show benefit in any of the variables evaluated. This strategy was also re-evaluated with modifications, i.e., induction chemotherapy (docetaxel, infusional FU, cisplatin) followed by neoadjuvant chemoradiation followed by surgery, with or without cetuximab [48]. The addition of cetuximab did improve the locoregional control but did not translate in survival benefit. With the availability of more evidence in the future, the strategy is worth watching for.

This metanalysis has its limitations. We have not separately analyzed the outcomes of adenocarcinoma and squamous cell carcinoma which are known to behave and respond differently to chemotherapy and chemoradiotherapy due to lack of clarity in reporting of outcomes for these subsets in the included trials. Also, the different chemotherapy regimens and different radiotherapy schedules and doses make the data heterogenous.

Having included all studies from 1981 to 2018, the long span of 37 years has diagnostic and therapeutic variations in the management of esophageal cancer ranging from differences in staging, chemotherapeutic agents, radiation technology, operative techniques, and critical care, making the interpretation of results even more challenging.

Additionally, we were not able to procure full texts of three trials that fulfilled eligibility criteria; hence, they were not included for the meta-analyses. Despite these shortcomings of the present meta-analysis, the results clearly indicate the benefits of neoadjuvant chemoradiation. With the availability of more data and more strategies, this too is set to change soon with induction chemotherapy followed by neoadjuvant chemoradiation followed by surgery emerging as viable and better option [49].

## Conclusions

This metanalysis shows unequivocal superiority of NACRT followed by surgery in the treatment of resectable esophageal cancer in terms of survival, and although this should be considered standard of care, it comes at the cost of significantly increased perioperative mortality. The role of chemotherapy in a purely neoadjuvant setting is questionable and if unavoidable, should be consolidated with adjuvant chemotherapy, after definitive surgery.

In light of the above facts, these results suggest the need for further research to determine the timing of chemoradiation to obtain maximum survival benefit without postoperative morbidity or mortality.

## Supplementary information

**Additional file1:.** Forest and funnel plot comparing 3-year DFS in NACRT and upfront surgery arms.

**Additional file 2:.** Forest and funnel plot comparing 5-year DFS in NACRT and upfront surgery arms.

**Additional file 3:.** Forest and funnel plot comparing perioperative mortality in NACT and upfront surgery arms.

**Additional file 4:.** Forest and funnel plot comparing failures in NACT and upfront surgery arms.

**Additional file 5:.** Forest and funnel plot comparing 3-year DFS in NACT and upfront surgery arms.

**Additional file 6:.** Forest and funnel plot comparing 3-year OS in NACT and upfront surgery arms.

**Additional file 7:.** Forest and funnel plot comparing 5-year DFS in NACT and upfront surgery arms.

**Additional file 8:.** Forest and funnel plot comparing 5-year OS in NACT and upfront surgery arms.

**Additional file 9:.** Forest and funnel plot comparing perioperative mortality in NART and upfront surgery arms.

**Additional file 10:.** Forest and funnel plot comparing failures in NART and upfront surgery arms.

**Additional file 11:.** Forest and funnel plot 3-year OS in NART and upfront surgery arms.

**Additional file 12:.** Forest and funnel plot comparing 5-year OS in NART and upfront surgery arms.

**Additional file 13:.** Forest and funnel plot comparing 3-year OS in SCRT and upfront surgery arms.

**Additional file 14:.** Forest and funnel plot comparing perioperative mortality in SCRT and upfront surgery arms.

**Additional file 15:.** PRISMA Checklist

## Data Availability

All data generated or analyzed during this study are included in this published article [and its supplementary information files].

## Abbreviations

CI: Confidence interval
DFS: Disease-free survival
EGC: Esophago-gastric cancer
FU: Fluorouracil
NACRT: Neoadjuvant chemoradiation
NACT: Neoadjuvant chemotherapy
NART: Neoadjuvant radiation
OR: Odds ratio
OS: Overall survival
PRISMA: Preferred Reporting Items for Systematic Reviews and Meta-Analyses
SCRT: Sequential chemoradiation
