# Supplementary figures and images for: Neoadjuvant strategies in resectable carcinoma esophagus: a meta-analysis of randomized trials
Source: World J Surg Oncol. 2020 Mar 21;18:59. doi: 10.1186/s12957-020-01830-x (PMC7085863; doi:10.1186/s12957-020-01830-x)

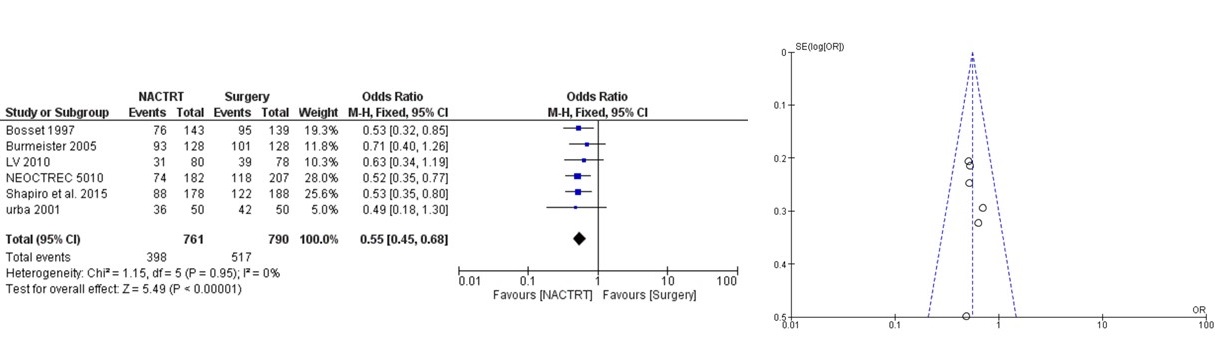

Supplement: Supplementary file 1 — Additional file1:. Forest and funnel plot comparing 3-year DFS in NACRT and upfront surgery arms. [file 12957_2020_1830_MOESM1_ESM.jpg]

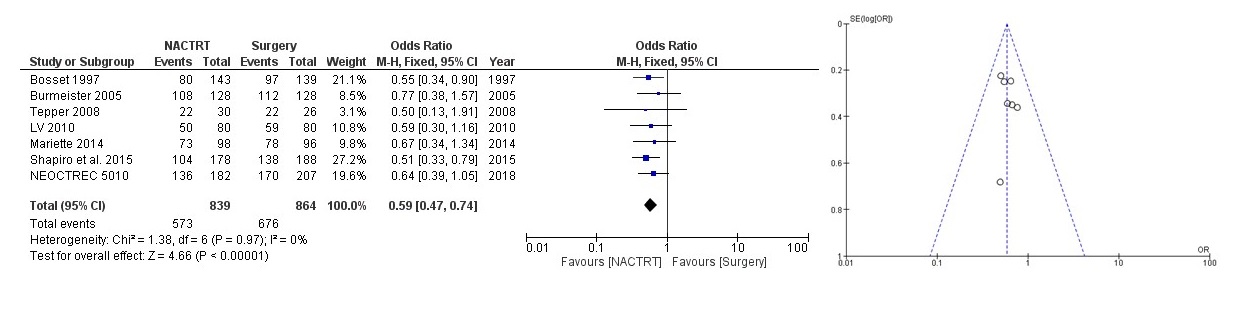

Supplement: Supplementary file 2 — Additional file 2:. Forest and funnel plot comparing 5-year DFS in NACRT and upfront surgery arms. [file 12957_2020_1830_MOESM2_ESM.jpg]

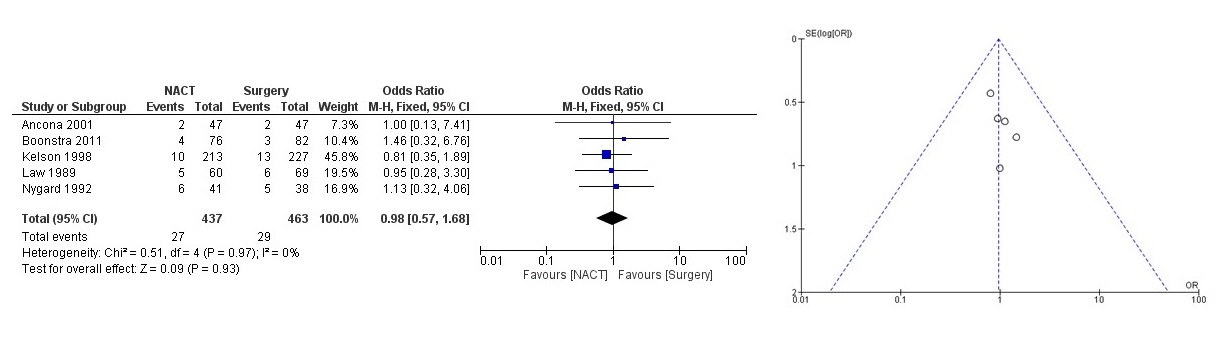

Supplement: Supplementary file 3 — Additional file 3:. Forest and funnel plot comparing perioperative mortality in NACT and upfront surgery arms. [file 12957_2020_1830_MOESM3_ESM.jpg]

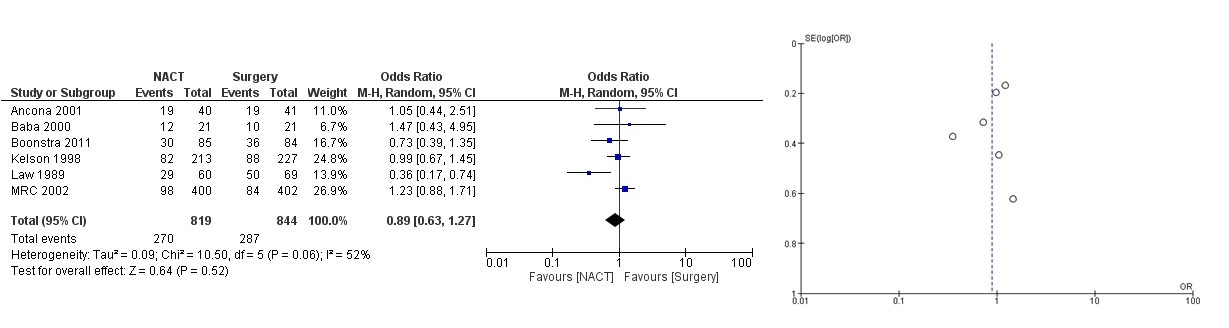

Supplement: Supplementary file 4 — Additional file 4:. Forest and funnel plot comparing failures in NACT and upfront surgery arms. [file 12957_2020_1830_MOESM4_ESM.jpg]

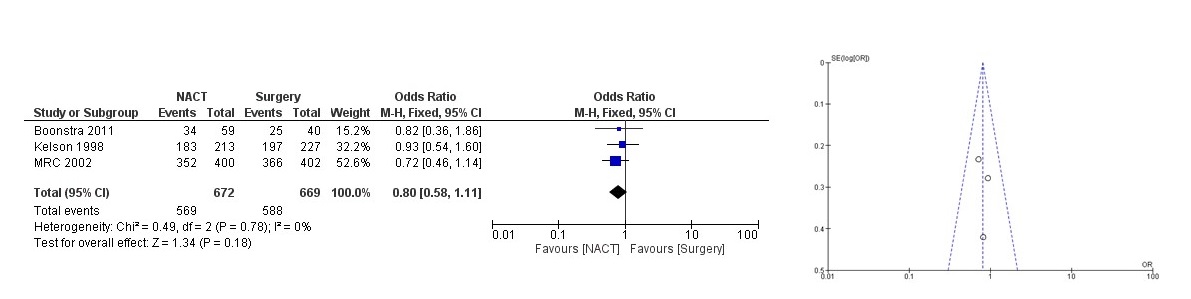

Supplement: Supplementary file 5 — Additional file 5:. Forest and funnel plot comparing 3-year DFS in NACT and upfront surgery arms. [file 12957_2020_1830_MOESM5_ESM.jpg]

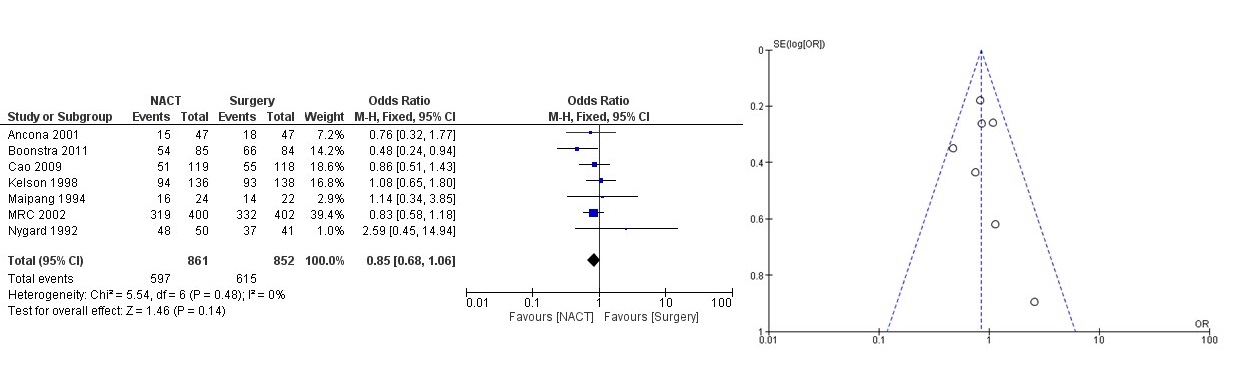

Supplement: Supplementary file 6 — Additional file 6:. Forest and funnel plot comparing 3-year OS in NACT and upfront surgery arms. [file 12957_2020_1830_MOESM6_ESM.jpg]

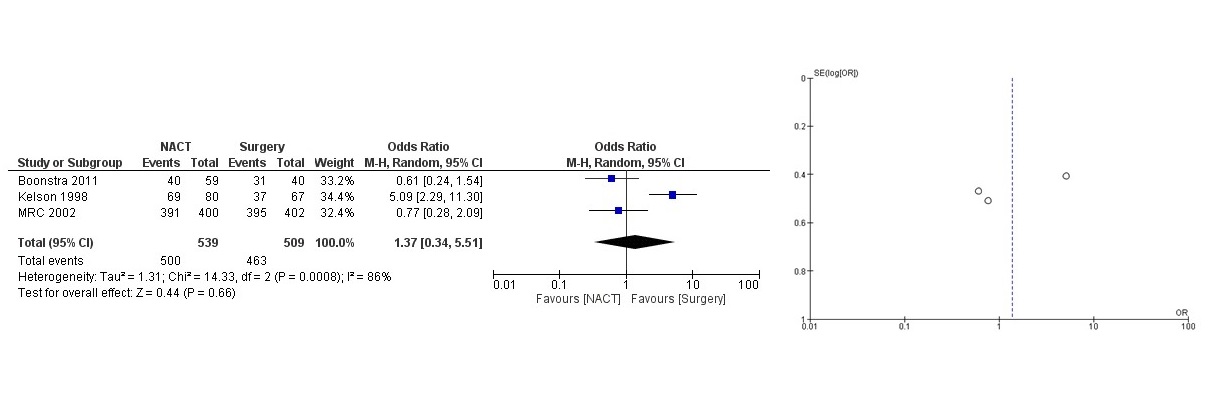

Supplement: Supplementary file 7 — Additional file 7:. Forest and funnel plot comparing 5-year DFS in NACT and upfront surgery arms. [file 12957_2020_1830_MOESM7_ESM.jpg]

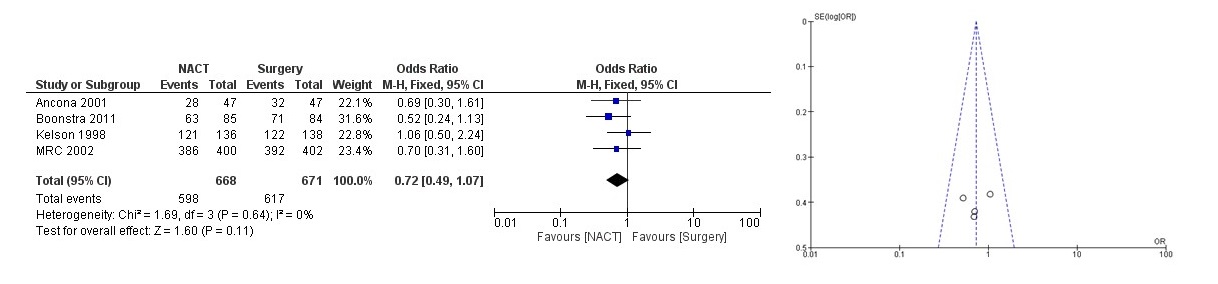

Supplement: Supplementary file 8 — Additional file 8:. Forest and funnel plot comparing 5-year OS in NACT and upfront surgery arms. [file 12957_2020_1830_MOESM8_ESM.jpg]

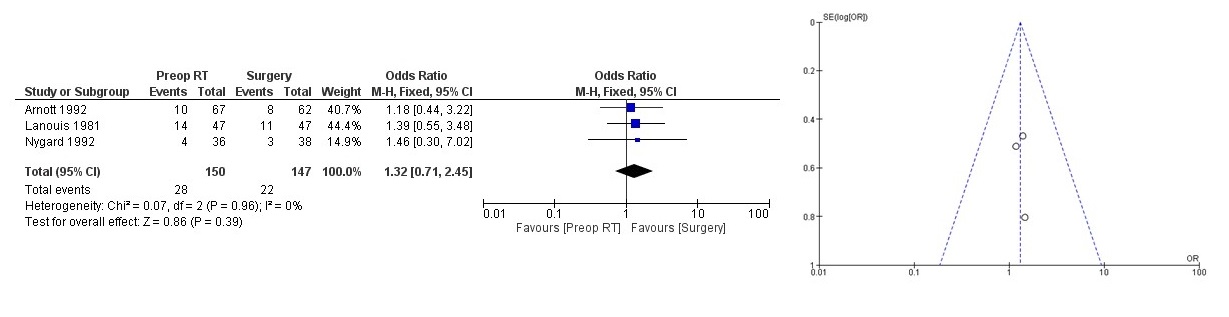

Supplement: Supplementary file 9 — Additional file 9:. Forest and funnel plot comparing perioperative mortality in NART and upfront surgery arms. [file 12957_2020_1830_MOESM9_ESM.jpg]

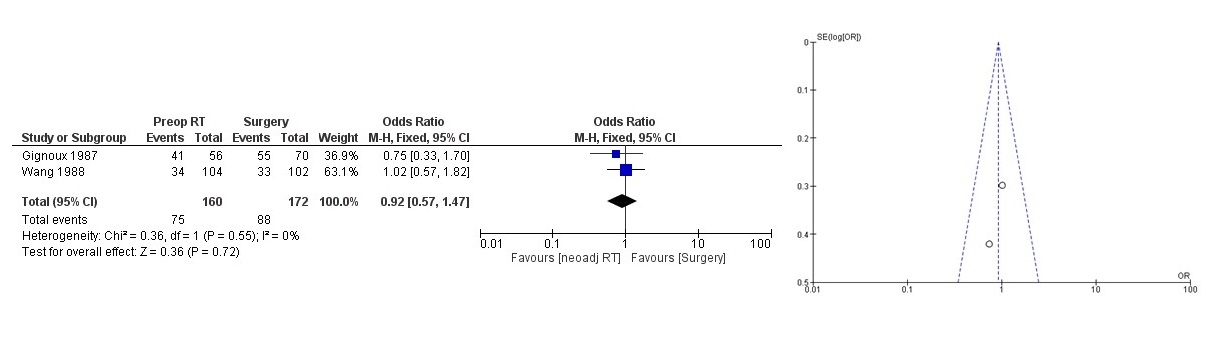

Supplement: Supplementary file 10 — Additional file 10:. Forest and funnel plot comparing failures in NART and upfront surgery arms. [file 12957_2020_1830_MOESM10_ESM.jpg]

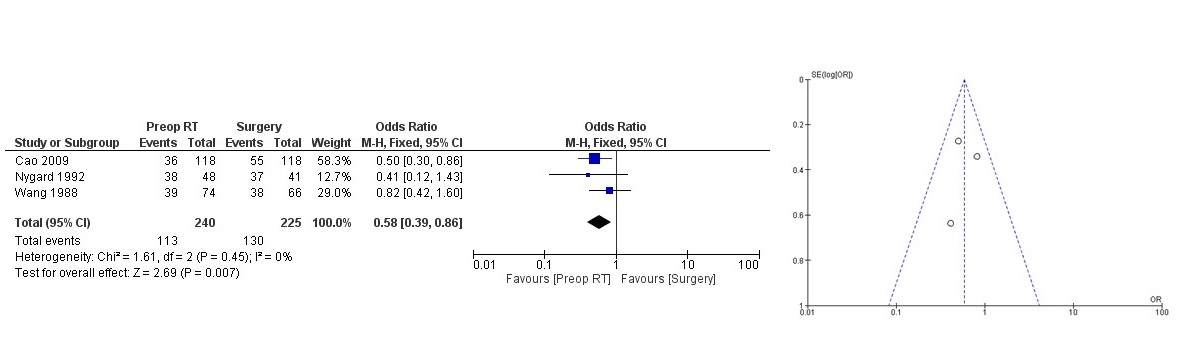

Supplement: Supplementary file 11 — Additional file 11:. Forest and funnel plot 3-year OS in NART and upfront surgery arms. [file 12957_2020_1830_MOESM11_ESM.jpg]

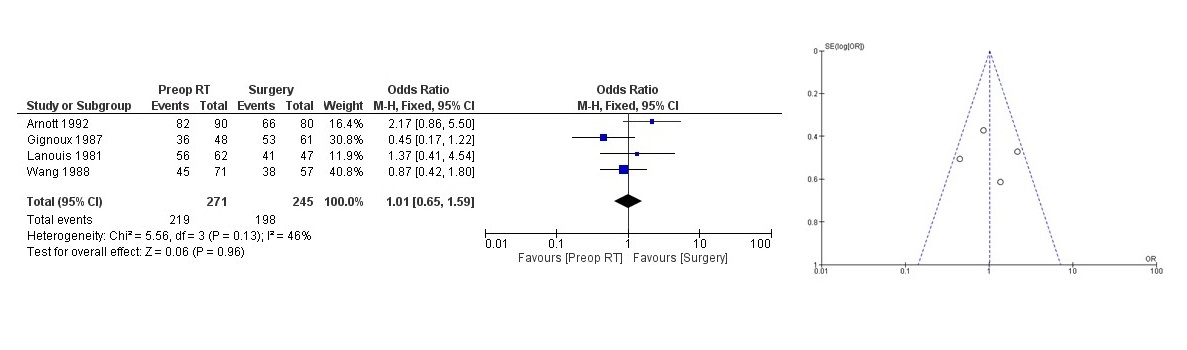

Supplement: Supplementary file 12 — Additional file 12:. Forest and funnel plot comparing 5-year OS in NART and upfront surgery arms. [file 12957_2020_1830_MOESM12_ESM.jpg]

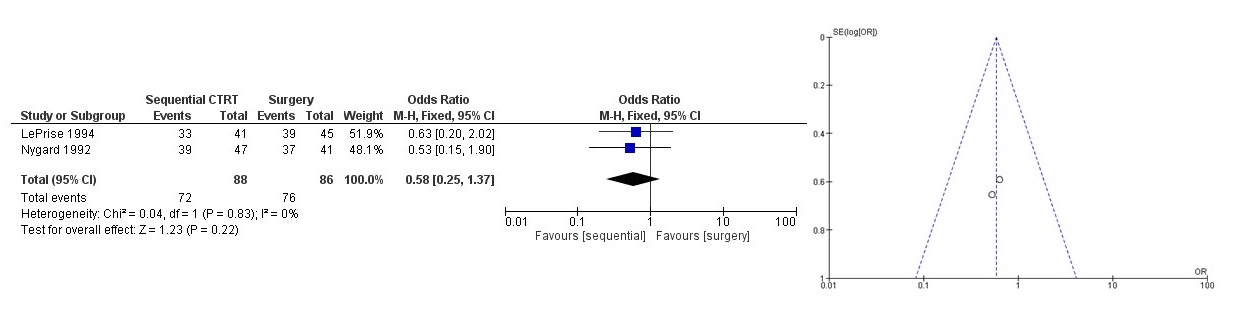

Supplement: Supplementary file 13 — Additional file 13:. Forest and funnel plot comparing 3-year OS in SCRT and upfront surgery arms. [file 12957_2020_1830_MOESM13_ESM.jpg]

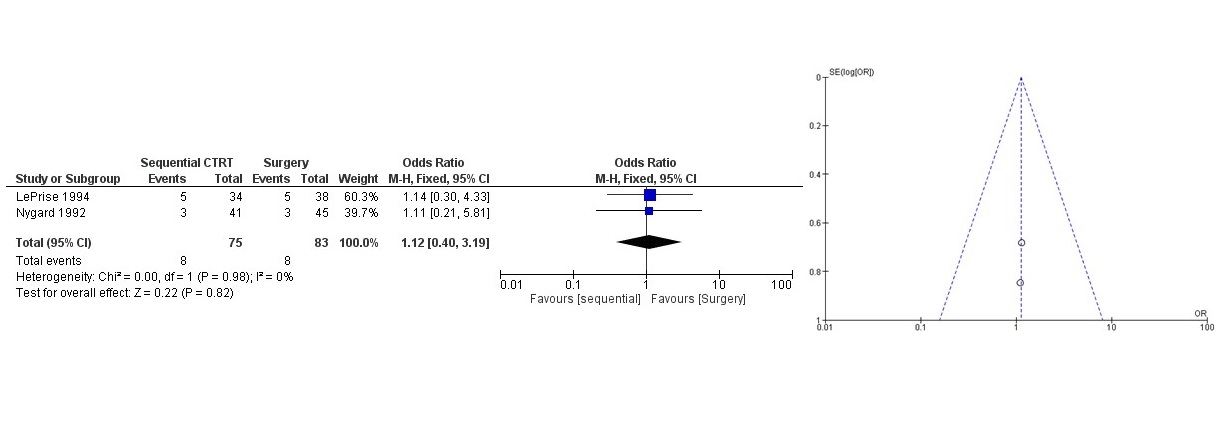

Supplement: Supplementary file 14 — Additional file 14:. Forest and funnel plot comparing perioperative mortality in SCRT and upfront surgery arms. [file 12957_2020_1830_MOESM14_ESM.jpg]
